# Supplementary material for: Splice-Junction-Based Mapping of Alternative Isoforms in the Human Proteome
Source: Cell Rep. Author manuscript; Available in PMC 2020 Jan 15. (PMC6961840; doi:10.1016/j.celrep.2019.11.026)
Supplement: 3 [file NIHMS1546469-supplement-3.zip › DF2/PXD006675/VentricularSeptum-19-Q14896-TDSHEDTGILDFSSLLK.pdf]

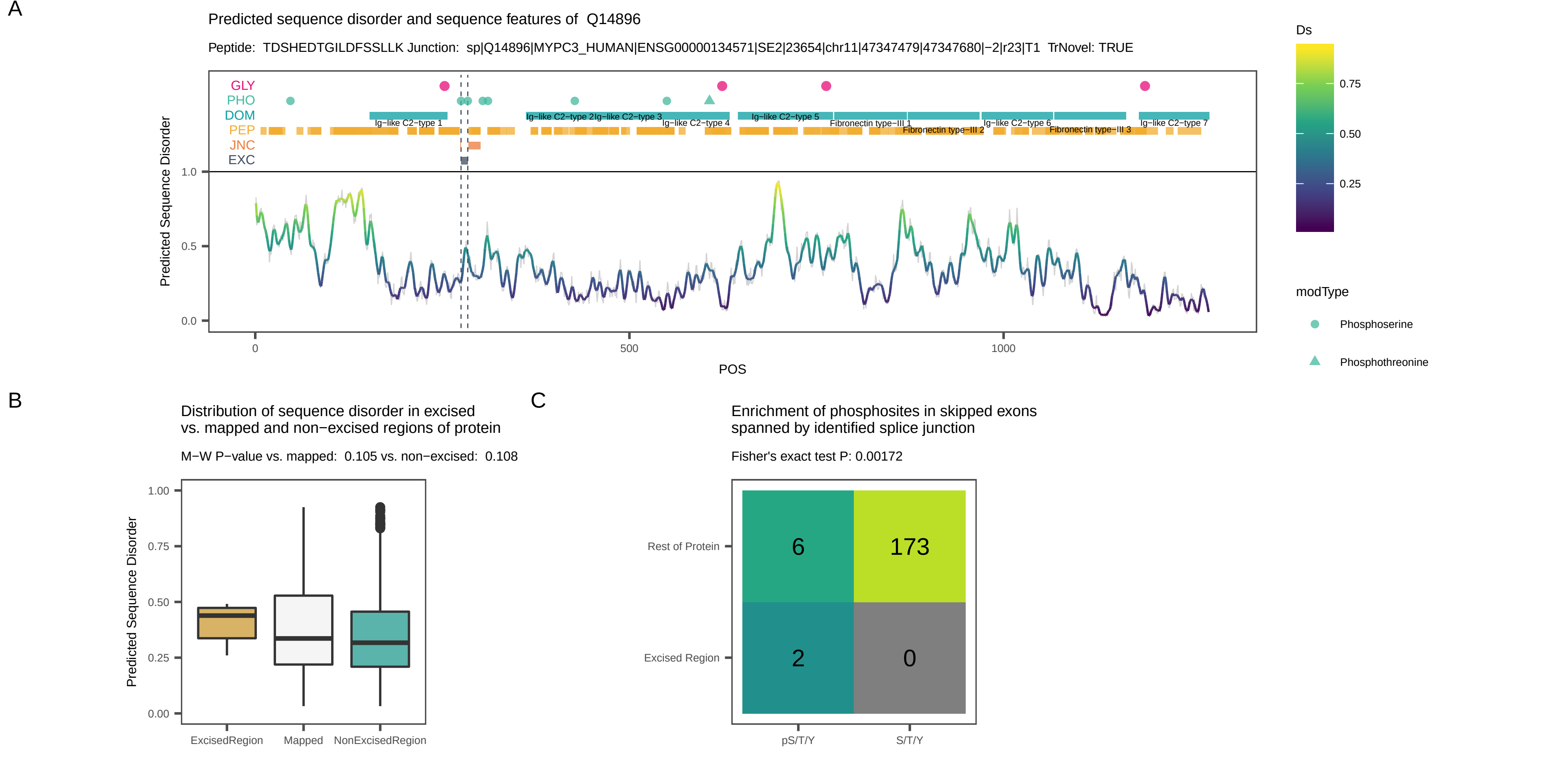

A
Predicted sequence disorder and sequence features of Q14896
Peptide: TDSHEDTGILDFSSLLK Junction: sp|Q14896|MYPC3_HUMAN|ENSG00000134571|SE2|23654|chr11|47347479|47347680|−2|r23|T1 TrNovel: TRUE
GLY
PHO
DOM
PEP
JNC
EXC
Ig−like C2−type 1
Ig−like C2−type 2
Ig−like C2−type 3
Ig−like C2−type 4
Ig−like C2−type 5
Fibronectin type−III 1
Fibronectin type−III 2
Ig−like C2−type 6
Fibronectin type−III 3
Ig−like C2−type 7
Predicted Sequence Disorder
POS
Ds
modType
Phosphoserine
Phosphothreonine
B
Distribution of sequence disorder in excised vs. mapped and non−excised regions of protein
M−W P−value vs. mapped: 0.105 vs. non−excised: 0.108
ExcisedRegion
Mapped
NonExcisedRegion
C
Enrichment of phosphosites in skipped exons spanned by identified splice junction
Fisher's exact test P: 0.00172
Rest of Protein
Excised Region
pS/T/Y
S/T/Y
6
173
2
0
